# Rituximab and Ocrelizumab in Multiple Sclerosis: Does Comparative Evidence Redefine Anti-CD20 Therapy?

**DOI:** 10.3390/jcm15218223

**Published:** 2026-10-22

**Authors:** Valentino Rački, Andrej Belančić, Almir Fajkić, Slobodan Janković

**Affiliations:** 1 Department of Neurology, Faculty of Medicine, University of Rijeka, 51000 Rijeka, Croatia; 2 Department of Basic and Clinical Pharmacology and Toxicology, Faculty of Medicine, University of Rijeka, 51000 Rijeka, Croatia; 3 Department of Pathophysiology, Faculty of Medicine, University of Sarajevo, 71000 Sarajevo, Bosnia and Herzegovina; 4 Faculty of Medical Sciences, University of Kragujevac, 34000 Kragujevac, Serbia

**Keywords:** rituximab, ocrelizumab, B-cell therapy, multiple sclerosis

## Abstract

B-cell depletion has transformed multiple sclerosis treatment, with rituximab and ocrelizumab providing highly effective CD20-directed therapy. This narrative review aims to critically compare these agents from the mechanistic, clinical, safety, regulatory, and economic perspectives. Despite their shared target, differences in pharmacology, dosing, regulatory status, and cost may influence treatment selection. Observational studies generally suggest similar effectiveness, although interpretation is limited by confounding and heterogeneous treatment strategies. The randomized, double-blind OVERLORD-MS trial provides the strongest direct evidence, demonstrating non-inferiority of rituximab to ocrelizumab for suppression of new or enlarging inflammatory lesions on magnetic resonance imaging (MRI) in newly diagnosed relapsing MS. However, evidence remains insufficient to establish equivalence for disability progression, long-term outcomes, or rare and cumulative adverse events. Uncertainty persists regarding infections, hypogammaglobulinemia (reduced immunoglobulin levels), pregnancy, optimal dosing, and long-term immune effects. Rituximab’s substantially lower cost could improve access to high-efficacy treatment; however, current evidence does not directly support assuming universal interchangeability with ocrelizumab.

## 1. Introduction

Multiple sclerosis (MS) is a chronic immune-mediated disease of the central nervous system characterized by inflammatory demyelination, neuroaxonal injury, and variable accumulation of neurological disability. Although MS was historically conceptualized predominantly as a T-cell-mediated disorder, its pathobiology reflects coordinated interactions among T cells, B cells, myeloid cells, and resident central nervous system immune populations. Autoreactive T cells contribute to inflammatory tissue injury, while B cells amplify disease through antigen presentation, co-stimulation, cytokine production, and antibody-associated mechanisms [1].

The recognition of B-cell contributions has been especially consequential therapeutically. Contemporary MS treatment ranges from escalation approaches to early use of high-efficacy therapies, with treatment intensity individualized according to disease activity, prognostic features, prior treatment response, comorbidity, reproductive plans, and access. Within this context, anti-CD20 monoclonal antibodies have become a central high-efficacy strategy.

Early clinical trial data with rituximab provided direct evidence for the therapeutic relevance of B-cell depletion. In relapsing-remitting MS, B-cell depletion was associated with a rapid reduction in inflammatory disease activity, at a pace that was difficult to explain solely by changes in pathogenic antibody concentrations [2]. This observation shifted attention toward antigen presentation and cellular crosstalk. It also created a practical question that has persisted ever since: if depletion of CD20-expressing cells is so effective, how much of the eventual treatment effect depends on the antibody used?

Rituximab became the first widely used anti-CD20 therapy in MS, although it was not developed through a dedicated licensing programme for the disease. Its adoption was driven by trial data, clinical experience, and large observational cohorts, particularly in settings where cost and access made it an attractive high-efficacy option [2,3]. Ocrelizumab followed a different path. It was developed specifically for MS and tested in phase 3 programmes in relapsing and primary progressive disease [4,5]. Those studies established ocrelizumab as a licensed anti-CD20 treatment and placed B-cell depletion near the centre of modern MS care.

This history is relevant for the current comparison as rituximab and ocrelizumab share the same target, but they entered MS practice through different evidence and regulatory routes. Ocrelizumab has a disease-specific development programme and labelled indication, while rituximab has a much longer period of off-label experience and, in several health systems, a markedly different economic position. Comparative evidence must therefore answer more than whether both agents suppress inflammatory activity. It must also clarify the limits of extrapolation across disease phenotypes, prior treatment exposure, safety domains, dosing approaches, and healthcare settings.

Although anti-CD20 therapy improved inflammatory disease control, it also highlighted the unresolved questions around durability, immune recovery, infection risk, treatment burden, and access.

It also changed the standard by which treatment studies are judged. A new anti-CD20 study is no longer assessed only against placebo or an older platform therapy. It is assessed against a class that has already altered expectations for inflammatory disease control. That makes direct comparative work valuable, but it also requires careful interpretation. Similar early control of inflammation does not settle every question that determines long-term treatment choice. This review therefore compares rituximab and ocrelizumab across mechanism, comparative efficacy, safety, treatment optimization, regulatory status, and health-economic implications, with particular attention to what the randomized comparative evidence establishes.

## 2. Methods

This article was designed as a narrative review to critically synthesize the available evidence comparing rituximab and ocrelizumab in multiple sclerosis, with emphasis on pharmacological characteristics, comparative efficacy, safety, treatment optimization, and economic and healthcare-access considerations. A narrative approach was considered appropriate given the breadth and heterogeneity of the available evidence, encompassing randomized clinical trials, observational and registry-based studies, pharmacological and mechanistic investigations, pharmacovigilance data, systematic reviews and meta-analyses, health-economic analyses, regulatory documents, and other relevant clinical literature.

A targeted literature search was conducted in PubMed (National Institute of Health, Bethesda, MD, USA) and Scopus (Elsevier, Amsterdam, The Netherlands), including publications available up to 20 August 2026. No restrictions were applied according to publication or study type. The search strategy combined terms related to the disease, interventions, and major domains of interest. Consequently, the core search terms included “multiple sclerosis”, “multiple sclerosis, relapsing-remitting”, “rituximab”, “ocrelizumab”, “anti-CD20”, “comparative effectiveness”, “efficacy”, “safety”, “adverse events”, “infection”, “immunoglobulin”, “hypogammaglobulinemia”, “B-cell depletion”, “dosing”, “extended interval dosing”, “treatment optimization”, “vaccination”, “pregnancy”, “pharmacoeconomics”, “cost-effectiveness”, “healthcare costs”, “reimbursement”, and “healthcare access”.

Studies were identified and screened by V.R. and A.B. Relevant publications were discussed within the author team, and S.J., as senior author, resolved potential discrepancies regarding relevance or interpretation. The multidisciplinary expertise of the authors in neurology, clinical pharmacology, evidence-based medicine, pharmacoeconomics, pathophysiology, and immunology supported the assessment of the clinical, pharmacological, biological, methodological, and economic aspects of the evidence.

Evidence was synthesized narratively and thematically. The relative strength and interpretability of findings were considered according to study design and methodological context, with randomized controlled trials receiving greater weight for direct comparative treatment effects, observational and registry studies informing real-world effectiveness and safety, and mechanistic, pharmacological, and economic studies interpreted according to their respective strengths and limitations. The synthesis was structured around pharmacological and mechanistic differences, comparative efficacy, safety, treatment optimization, and economic, regulatory, and healthcare-access considerations.

As a narrative review, no formal systematic-review protocol, quantitative meta-analysis, or formal risk-of-bias assessment was undertaken. Instead, the strength and limitations of individual findings were interpreted in the context of study design, directness of comparison, potential confounding, endpoint characteristics, follow-up duration, and applicability to different multiple sclerosis populations and treatment settings.

## 3. Pharmacological Similarities and Differences

Rituximab and ocrelizumab share the same therapeutic target, but they are structurally and pharmacologically distinct antibodies. Rituximab is a chimeric (mouse–human) IgG1 monoclonal antibody, whereas ocrelizumab is a humanized IgG1 antibody derived from the 2H7 lineage [6,7]. Both are type I anti-CD20 antibodies and recognize the large extracellular loop of CD20. Their binding sites overlap, but structural studies show differences in the precise way each antibody interacts with CD20 [6,7]. Humanization of ocrelizumab reduced the proportion of non-human sequence while retaining CD20 recognition, a modification intended to reduce immunogenicity without compromising target engagement.

CD20, encoded by MS4A1, is a membrane-spanning protein expressed from the pre-B-cell stage through mature and memory B cells, while being absent from hematopoietic stem cells and largely lost during terminal differentiation into plasma cells [8]. This expression pattern allows anti-CD20 antibodies to produce profound B-cell depletion while preserving the capacity for B-cell regeneration and largely sparing long-lived plasma cells. Their effects in multiple sclerosis (MS), however, extend beyond simple removal of circulating B cells. B cells participate in antigen presentation, T-cell co-stimulation, and cytokine production, and rituximab-mediated B-cell depletion has been associated with attenuation of pro-inflammatory Th1 and Th17 responses [1]. CD20 is also expressed by a small population of T lymphocytes: CD3+CD20dim T cells with pro-inflammatory features are efficiently depleted by rituximab [9], while ocrelizumab similarly produces rapid depletion of circulating CD3+CD20+ T cells [10]. Anti-CD20 therapy should therefore be viewed as modulation of a CD20-expressing lymphocyte compartment rather than exclusively as B-cell depletion. CD20 is not a disease-specific antigen and is not known to be uniquely overexpressed in MS; rather, it provides a therapeutically accessible marker on B-cell and small T-cell populations that participate in MS immunopathology. This distinction is important when interpreting anti-CD20 therapy as selective depletion of a pathogenic immune compartment rather than correction of a CD20-specific molecular abnormality.

Binding to the same target does not imply identical downstream effector activity. Rituximab and ocrelizumab can both recruit antibody-dependent cellular cytotoxicity (ADCC), antibody-dependent cellular phagocytosis, complement-dependent cytotoxicity (CDC), and direct cell-death pathways [6]. Their relative contributions differ, however. Ocrelizumab has greater ADCC and lower CDC activity than rituximab in vitro and shows enhanced binding to the low-affinity FcγRIIIa variant [6]. Rituximab, conversely, relies more strongly on complement-mediated cytotoxicity. These properties offer a mechanistic basis for potentially different depletion kinetics or treatment-related effects, although their clinical importance cannot be inferred directly from in vitro potency. Fc-receptor genotype, CD20 density, complement availability, tissue penetration, and repeated exposure all modify antibody activity in vivo.

The pharmacology of ocrelizumab has been characterized systematically during its MS development programme. Its concentration–time profile is described by a two-compartment model with both constant and time-dependent clearance [11]. The estimated terminal half-life is approximately 26 days, and body weight is the principal covariate influencing systemic exposure [11]. Peripheral CD19+ B-cell counts are reduced by day 14 after infusion [12]. Pharmacodynamic recovery is considerably slower than antibody elimination: among 51 adults followed after discontinuation, the median time for B-cell counts to return to baseline or the lower limit of normal was 72 weeks [12]. This separation between serum pharmacokinetics and B-cell reconstitution is important because it provides a biological rationale for dosing strategies based on sustained pharmacodynamic activity rather than serum drug persistence alone.

Rituximab has not undergone an equivalent MS-specific pharmacological development programme and has no licensed MS dosing regimen [13]. In HERMES, two 1000-mg infusions on days 1 and 15 reduced peripheral CD19+ B cells by more than 95% from weeks 2 through 24, with repopulation becoming evident thereafter [2]. In clinical practice, lower doses and longer retreatment intervals have also been used; a large Swedish cohort, for example, commonly employed 500- or 1000-mg infusions every 6–12 months [3]. Differences between the two drugs observed in practice may therefore reflect not only intrinsic antibody properties but also exposure, treatment interval, and the duration of B-cell suppression.

Immunogenicity is another pharmacologically relevant distinction. The murine component of rituximab provides a greater theoretical substrate for anti-drug immune responses than the humanized structure of ocrelizumab. In HERMES, human anti-chimeric antibodies were detected in 24.1% (14/58) of evaluable rituximab-treated participants by week 48 [2]. In OLYMPUS, human anti-chimeric antibody positivity occurred in 7.0% (20/286) of rituximab-treated participants, although positivity was also detected in 6.3% (9/143) of placebo recipients [14]. By comparison, anti-drug antibodies were reported in 12 of 1131 (~1%) ocrelizumab-treated adults in the current US prescribing information, including neutralizing antibodies in two patients [12] (Table 1). These percentages should not be interpreted as a direct comparison: assay sensitivity and specificity, sampling schedules, drug interference, exposure, and definitions of positivity differ substantially between development programmes. Nevertheless, the contrast illustrates why antibody humanization may be pharmacologically relevant even when its clinical consequences remain uncertain.

The regulatory trajectories of the two antibodies ultimately diverged. Rituximab remains an off-label treatment for MS despite extensive clinical experience [3,15,16,17,18,19,20,21], whereas ocrelizumab underwent a dedicated MS development programme and is licensed for relapsing forms of MS and primary progressive MS [8]. The pivotal OPERA I/II and ORATORIO programmes provided the clinical evidence underpinning its MS development and regulatory pathway; their efficacy findings are considered in detail in the following section (Table 1). Ocrelizumab therefore carries an MS-specific indication, dosing framework, and regulatory evidence package that rituximab does not.

Rituximab and ocrelizumab should thus be regarded as closely related but pharmacologically non-identical approaches to CD20 depletion. Their molecular architecture, balance of Fc-mediated effector functions, pharmacokinetics, B-cell reconstitution, and immunogenic potential differ in biologically relevant ways. Whether these differences are sufficiently large to influence efficacy or safety in patients, however, cannot be determined from pharmacology alone and requires direct comparative clinical evidence.

## 4. Evidence Before the Randomized Comparison

Before a direct randomized comparison became available, rituximab and ocrelizumab were compared through a heterogeneous set of studies that answered related, but not identical, questions (Table 2). The pivotal trials established that both agents had important activity in MS. They did not establish that the agents were interchangeable.

Rituximab first showed proof of principle in the HERMES trial, a placebo-controlled study in relapsing-remitting MS [2]. The findings were clinically influential because depletion of CD20-expressing cells produced a prompt reduction in inflammatory activity. HERMES did not compare rituximab with another anti-CD20 antibody, nor was it designed to address the longer-term questions that are current in routine practice: cumulative immune effects, treatment sequencing, dose intensity, vaccine response, pregnancy planning, and rare adverse outcomes.

Ocrelizumab was evaluated in a broader MS-specific programme. OPERA I and OPERA II compared ocrelizumab with interferon beta-1a in relapsing disease, while ORATORIO compared ocrelizumab with placebo in primary progressive MS [4,5]. These trials established efficacy against their assigned comparators and supplied the regulatory foundation for ocrelizumab. They should not, however, be read as indirect proof of superiority over rituximab. Trial populations, comparators, calendar periods, outcome definitions, MRI acquisition, background standards of care, and follow-up all differ. Cross-trial ranking is especially unreliable when event rates are low or when the clinical question concerns a population that was not enrolled in the original studies.

The evidence base in primary progressive MS (PPMS) is asymmetric. In OLYMPUS, rituximab did not significantly reduce the primary endpoint of 12-week confirmed disability progression in the overall PPMS population, whereas ORATORIO demonstrated a significant reduction in confirmed disability progression with ocrelizumab and supported its licensed PPMS indication [5,14]. Randomized comparative evidence from relapsing MS should therefore not be extrapolated to therapeutic equivalence in PPMS.

Registries and real-world cohorts therefore provided additional comparative evidence. Their main strength is relevance. They describe treatments as prescribed, including off-label rituximab use, different infusion doses, extended intervals, prior treatment exposure, and the constraints imposed by local reimbursement policies. They can also provide larger samples and longer follow-up than an individual trial. Swedish data were important in establishing the scale of rituximab use in MS and in describing its effectiveness and safety in clinical practice [3].

Direct comparative evidence then accumulated across several settings rather than through one definitive cohort. The multinational registry analysis by Roos and colleagues was an important early direct comparison [15]. It was followed by a regional cohort from British Columbia, the 2025 Acta Neurologica Belgica study, and a Finnish population-based cohort [16,17,18]. These studies are useful because they examine the question in different prescribing environments, but their inclusion criteria, baseline treatment history, rituximab regimens, MRI availability, follow-up, and analytic approaches differ. Comparative safety studies have addressed infection or broader safety profiles rather than assuming that an effectiveness estimate also answers a safety question [19,20]. The ROC-MS collaboration adds a planned prospective individual-participant-data meta-analysis of four randomized trials, but its pooled results are not yet available [22].

The weakness of this evidence is the lack of randomization. A patient offered ocrelizumab may differ from a patient offered rituximab in ways that are only partly visible in a registry. Clinicians may choose one agent because of disease activity, previous treatment, age, reproductive plans, comorbidity, infusion infrastructure, insurance status, or local policy. Dose and interval also vary more for rituximab than for ocrelizumab. Even careful propensity methods can only adjust for measured factors and cannot fully remove confounding by indication.

Comparisons are also complicated by differences in MRI frequency and acquisition protocols. Relapses are subject to diagnostic and reporting variation, and disability measures may be missing or collected at different intervals. Safety comparisons are particularly vulnerable to unequal exposure time, surveillance intensity, prior immunosuppression, and incomplete capture of events outside the treatment centre. A lower observed event rate is therefore not necessarily evidence of lower biological risk [19].

A further limitation is time. Rituximab cohorts often represent a long period of clinical use, during which MRI access, diagnostic criteria, treatment thresholds, and background standards changed. Ocrelizumab cohorts are commonly more recent and may have shorter follow-up. Calendar time can therefore become an unmeasured treatment characteristic. It may influence surveillance, referral patterns, vaccination practices, and the probability that a complication is recorded. The same issue applies to dose. Rituximab is not administered in one universally accepted MS regimen. Some cohorts use induction followed by lower maintenance doses; others use repeated higher doses or extend the interval after stable disease. Ocrelizumab is more often delivered according to its approved schedule. If outcomes appear similar, the finding may reflect several successful rituximab strategies rather than a single interchangeable regimen. If outcomes differ, the reason may be dose intensity or patient selection rather than the antibody itself.

Direct comparative cohorts can still be highly informative when they are designed carefully. Matching or weighting can improve balance in measured baseline factors, and sensitivity analyses can show whether the estimate is robust to plausible analytic choices. These methods do not create randomization. They do, however, make the remaining uncertainty easier to see. A review should give that uncertainty the same prominence as the point estimate.

The recent randomized head-to-head trial changes the hierarchy of evidence for newly diagnosed relapsing MS, but it does not replace the earlier evidence base [23]. The earlier trials explain why anti-CD20 therapy became standard high-efficacy treatment. Registries explain how rituximab and ocrelizumab have been used outside trial protocols. Together they define the context in which a non-inferiority result should be read: as stronger evidence for a specific comparative question, not as a general conclusion for every patient, dose, disease phenotype, or safety outcome.

## 5. Randomized Comparative Evidence: The OVERLORD-MS Trial

For more than a decade, comparative effectiveness between rituximab and ocrelizumab relied almost exclusively on observational cohorts, registry studies, and indirect comparisons, all of which were inherently susceptible to residual confounding, treatment-selection bias, heterogeneous dosing strategies, and differences in monitoring protocols. The publication of the OVERLORD-MS trial in 2026 provided the first randomized, double-blind, head-to-head evidence comparing rituximab and ocrelizumab, substantially strengthening the evidence base beyond observational comparative studies.

OVERLORD-MS was an investigator-initiated, phase 3, multicentre, randomized, double-blind, active-controlled non-inferiority trial conducted at 12 centres in Norway and Sweden. It enrolled adults aged 18–60 years with relapsing MS diagnosed within the preceding 12 months, recent inflammatory disease activity, EDSS 0–4.0, and no previous disease-modifying therapy. Participants were randomized 3:2 to rituximab (1000 mg initially, then 500 mg every six months) or ocrelizumab (600 mg every six months) [23].

The prespecified primary endpoint was freedom from new or enlarging T2-weighted MRI lesions between months 6 and 24. Non-inferiority was assessed for the absolute risk difference (rituximab minus ocrelizumab) against a −10-percentage-point margin. The trial was powered for this MRI endpoint, not for equivalence or non-inferiority of relapse, disability, or safety outcomes [23]. Among 218 randomized participants, 216 received at least one infusion (132 rituximab and 84 ocrelizumab), and 93% completed the 30-month follow-up. The probability of remaining free from new or enlarging T2-weighted lesions between months 6 and 24 was 92.2% with rituximab and 94.8% with ocrelizumab, an absolute risk difference of −2.6 percentage points (95% CI −9.4 to 4.3). Because the lower confidence limit remained above the prespecified −10-percentage-point margin, rituximab met the non-inferiority criterion. The lower confidence bound was nevertheless close to that margin, so the result supports non-inferiority only for the prespecified MRI outcome and should not be interpreted as therapeutic equivalence [23].

Secondary outcomes were directionally similar, with very low annualized relapse rates (0.09 with rituximab versus 0.04 with ocrelizumab), high relapse-free survival, and no clear between-group difference in disability or cognitive outcomes. These analyses were secondary and the trial was not powered to establish equivalence for clinical outcomes or safety [23]. Randomization, double blinding, centralized MRI interpretation, independent relapse adjudication, certified EDSS assessment, and prespecified analyses strengthen the internal validity of the MRI comparison [23].

The principal limitations are equally important. The primary endpoint measured MRI inflammatory activity rather than disability accumulation; the clinical and safety analyses were underpowered; 30 months is insufficient to assess cumulative hypogammaglobulinemia, malignancy, or long-term disability; and external validity is constrained by a treatment-naïve, early relapsing population recruited mainly in Norway and Sweden. Applicability to previously treated patients, progressive MS, more diverse populations, and alternative dosing regimens therefore remains uncertain [23].

OVERLORD-MS therefore changes the hierarchy of evidence without closing the comparative question. It provides randomized support for rituximab non-inferiority to ocrelizumab for suppression of new or enlarging T2-weighted lesions in treatment-naïve early relapsing MS, but it does not establish equivalence across clinical efficacy, safety, disease phenotypes, or treatment strategies [23].

## 6. Comparative Safety and Remaining Uncertainties

Rituximab and ocrelizumab have often been compared in terms of efficacy and cost, whereas direct safety comparisons are less mature. The two antibodies differ in epitope interactions and in the relative contribution of complement- and antibody-dependent effector mechanisms, but pharmacological differences alone cannot establish clinically meaningful differences in safety.

OVERLORD-MS provides the only published randomized, double-blind head-to-head safety data (Table 3). Among 216 treated participants, serious adverse events were similar between groups (8% with rituximab and 7% with ocrelizumab), whereas at least one infection was reported in 82% of rituximab-treated and 69% of ocrelizumab-treated participants [23].

The between-group difference in overall infections is an observed finding from a double-blind trial and should not be dismissed as ascertainment bias. However, the study was powered for MRI non-inferiority rather than safety, the ocrelizumab arm included only 84 treated participants, and 30 months is insufficient to compare rare or cumulative harms. The small difference in hypogammaglobulinemia (0% with rituximab versus 2% with ocrelizumab) is similarly too imprecise to establish a molecule-specific advantage. OVERLORD-MS therefore informs short-term comparative safety but does not establish safety equivalence [23].

Observational comparisons are larger and longer but inconsistent. In the MSBase and Danish registry analysis, rituximab was associated with more relapses, although disability accumulation did not differ [15]. In the University of California cohorts, rituximab was associated with higher all-cause hospitalization and a greater risk of hypogammaglobulinemia [20]. By contrast, a nationwide Swedish study found similar infection and relapse risks with the two agents [19], and a Finnish matched cohort likewise reported comparable effectiveness and safety [17]. Differences in patient selection, dosing, prior immunosuppression, follow-up duration, healthcare utilization, and outcome ascertainment may contribute to this heterogeneity, but observational data cannot determine the contribution of each factor [15,17,19,20].

### 6.1. Specific Safety Considerations

#### 6.1.1. Infusion-Related Reactions (IRRs)

In a study comparing infusion-related reactions after switching from rituximab to ocrelizumab (100 patients switched and 100 remained on rituximab; 24-week follow-up), reactions were similar in frequency (14% in each group) and severity, with none severe enough to require hospitalization. With ocrelizumab, infusion-reaction risk was higher when circulating CD19 and/or CD20 cells were detectable (≥1%) [26]. A pharmacovigilance analysis of the FDA Adverse Event Reporting System (FAERS) likewise did not identify a clear difference in the proportion of infusion-reaction reports between rituximab and ocrelizumab [27].

#### 6.1.2. Infections

Infection risk is likely a class effect of B-cell-depleting anti-CD20 therapy. OVERLORD-MS reported more participants with at least one infection in the rituximab group, while serious events were uncommon [23]; the nationwide Swedish analysis found no clear difference in infection risk between rituximab and ocrelizumab [19]. In FAERS, infections accounted for 21.93% of ocrelizumab reports and 11.05% of rituximab reports [27]. These percentages are proportions of spontaneous adverse-event reports, not incidence rates or comparative risks, and they are vulnerable to differential reporting and exposure denominators.

#### 6.1.3. Hypogammaglobulinemia

Hypogammaglobulinemia is a cumulative, exposure-duration-dependent phenomenon. Real-world studies and systematic reviews generally indicate more frequent IgG decline with rituximab, although estimates vary across cohorts and dosing strategies [20,24,25]. Whether treatment-related hypogammaglobulinemia independently predicts clinically important infection is not fully resolved; baseline and periodic IgG measurement is therefore reasonable with either agent [25,35].

#### 6.1.4. Malignancy

No reliable differential signal exists. A single malignant melanoma occurred in the ocrelizumab arm of OVERLORD-MS [23]. The breast cancer imbalance seen in the ocrelizumab clinical trial when compared to interferon beta-1a (0.5% vs. 0.2%) in patients with RMS [4,28] has not been confirmed in extended follow-up, and no comparative study to date accumulates sufficient exposure time to address this endpoint. A few case reports point to increased risk of malignancy in general when ocrelizumab and other CD20-depleting monoclonal antibodies are used in RMS patients, but without a denominator (exact population exposed) this risk cannot be confirmed for the time being [29]. The pooled prevalence of malignancies when rituximab was used for treatment of multiple sclerosis was estimated on 0.39% after systematic review of 27 studies [30], but again, studies with larger samples and longer follow-up are necessary to confirm or reject increased risk of malignancy with CD20-depleting therapy.

#### 6.1.5. Pregnancy

Both agents are IgG1 antibodies and can cross the placenta, particularly during the second and third trimesters, creating a potential for transient neonatal B-cell depletion. Prospective MS-specific data are more developed for ocrelizumab: MINORE and SOPRANINO found low infant exposure and preserved infant B-cell counts, although exposed samples remained modest [31], and additional MS pregnancy cohorts are available [32]. Rituximab pregnancy evidence historically relied heavily on hematological and rheumatological populations [33], but MS-specific data also exist: a cohort of 74 pregnancies among 55 women treated with rituximab for MS did not identify a major congenital-malformation signal or an association between infusion timing/cumulative dose and miscarriage, while remaining observational and underpowered for rare outcomes [34]. Regulatory advice differs between agents: the current Ocrevus SmPC recommends contraception during treatment and for 4 months after the last dose [8], whereas the MabThera SmPC recommends effective contraception during treatment and for 12 months afterward [13]. A single washout rule should therefore not be applied to both drugs.

#### 6.1.6. Vaccination

Both agents attenuate humoral responses to non-live vaccines, although cellular responses may be relatively preserved [36,37]. Current regulatory guidance should be distinguished by agent. For ocrelizumab, required immunisations should be completed at least 6 weeks before treatment initiation; live or live-attenuated vaccines are not recommended during treatment and until B-cell repletion, and live vaccines in infants exposed in utero should be delayed until B-cell recovery [8]. For rituximab, vaccination status should be reviewed before therapy, vaccinations should be completed at least 4 weeks before the first dose where possible, and live viral vaccines are not recommended during treatment or while patients remain peripherally B-cell depleted [13].

#### 6.1.7. Long-Term Safety

Long-term comparative safety remains incompletely characterized. The nationwide Swedish analysis provides several years of follow-up (mean 5.4 years for rituximab and 4.7 years for ocrelizumab) [19], while other direct comparative cohorts have shorter exposure [17,20]. These data improve precision for common infections but still provide insufficient event numbers and duration to resolve rare malignancies, opportunistic infections, or cumulative immune effects. Extended-interval rituximab studies address dosing durability rather than a direct long-term safety comparison [38].

On present evidence, the short-term safety profiles of rituximab and ocrelizumab overlap substantially, but “comparable” is not synonymous with “interchangeable.” OVERLORD-MS was not powered for safety equivalence, observational findings remain inconsistent, and the pregnancy evidence base and regulatory guidance differ. ROC-MS is a planned prospective individual-participant-data meta-analysis of four randomized trials (OVERLORD-MS, DanNORMS, Noisy Rebels, and TRIO), intended to pool data from 1109 participants, with results expected in 2028 [22]. Until those pooled data and longer follow-up are available, treatment choice should rely on indication, regulatory status, patient-specific risk, and systematic monitoring rather than an assumption of safety equivalence.

## 7. Treatment Optimization in Clinical Practice

Anti-CD20 treatment is commonly administered at fixed intervals after depletion of circulating B cells, while in practice there are numerous differences between patients and between centres. Rituximab is used with more variation in dose and re-treatment schedule, while ocrelizumab is usually given according to a licensed six-month regimen, although extended interval dosing is being increasingly used. This difference matters because a comparison between drugs can easily become a comparison between treatment strategies.

### 7.1. Extended-Interval Dosing

Extended-interval dosing has attracted interest for practical and biological reasons. Some centres delayed infusions during the COVID-19 pandemic, while others explored longer intervals to reduce treatment burden, allow vaccine responses to recover, or limit cumulative immune suppression. Observational studies of ocrelizumab and rituximab have reported clinical and radiological outcomes under extended intervals [38,39,40,41]. These data are useful, but they should not be treated as proof that a particular interval is safe for every patient. Definitions of an “extended” interval differ, as do baseline disease activity, prior treatment, MRI surveillance, and the reason an infusion was delayed. Patients selected for interval extension may already have had stable disease.

### 7.2. B-Cell-Guided Retreatment

Peripheral B-cell monitoring is biologically appealing but has not yet become a universally validated redosing rule. CD19-positive cell recovery can show that depletion is no longer complete, yet repopulation is heterogeneous and does not map directly onto an individual risk of relapse or new MRI activity [41]. We cannot assess directly which subsets of B-cells have returned, how they relate to tissue-level immune activity, and whether they improve prediction beyond clinical history and MRI.

For now, B-cell counts are best viewed as pharmacodynamic information rather than a stand-alone treatment target. They can inform a discussion about interval length, vaccination timing, or unexpected immune recovery, but they cannot replace clinical assessment and MRI surveillance. Prospective studies need to test biomarker-guided dosing against fixed-interval treatment rather than infer clinical utility from a correlation observed in a cohort.

### 7.3. De-Escalation and Switching

Another important part of treatment optimization is de-escalation, which is not clearly defined yet. It can mean reducing a treatment dose, extending an infusion interval, temporarily withholding treatment, stopping therapy, or changing to another disease-modifying treatment. Those decisions have different risks. A patient with sustained disease control, recurrent infection, low immunoglobulin concentrations, pregnancy plans, or limited treatment access may raise a different question from a patient with recent inflammatory activity. The record should make the rationale explicit instead of treating all less-intensive approaches as equivalent.

Switching deserves the same caution. Transition from fingolimod to rituximab or ocrelizumab has been studied in relation to early and later treatment response, but washout duration, prior disease activity, and treatment selection can shape the result [42]. Evidence on direct rituximab-to-ocrelizumab switching is more limited and should not be assumed from class effects. When a switch is considered, the practical issues are usually as important as the theoretical ones: current disease activity, infusion timing, immune status, pregnancy plans, vaccination needs, prior adverse events, and the reason for changing treatment.

### 7.4. Special Populations and Monitoring

Special populations highlight the limitations of applying a uniform treatment schedule. Pregnancy and infant outcome studies, pediatric-onset multiple sclerosis (POMS) cohorts, older patients, and people with comorbidity are not interchangeable with the populations enrolled in pivotal trials [32,43]. Their evidence base is often observational and can be incomplete, and standard treatment schedules may not have the same implications across all circumstances. Treatment optimization should therefore be framed as structured uncertainty: use the best evidence available, state where it is indirect, and avoid turning a local protocol into a universal rule.

Furthermore, monitoring also has to distinguish purpose from habit. A full blood count, immunoglobulin measurement, infection history, MRI result, and B-cell measurement do not answer the same question. Ordering them as a fixed panel can give an impression of precision without a defined action threshold. A more useful approach is to state in advance what result would change the plan: closer follow-up, vaccination before the next infusion, a longer interval, referral for immunological assessment, or a change in treatment. This is particularly important when long-term exposure accumulates and when risks may be influenced by treatments received before anti-CD20 therapy.

Finally, there is also a practical dimension that affects everyday clinic work. A schedule that works in a tertiary MS center with reliable MRI access and prompt laboratory review may be less safe in a setting where follow-up is irregular. Conversely, a rigid six-month infusion pattern may create avoidable barriers for patients who travel long distances, have caregiving responsibilities, or require coordinated vaccination and pregnancy planning. The right strategy is not necessarily the most intensive one. It is the strategy whose benefits, uncertainties, and monitoring demands can be delivered consistently.

## 8. Economics, Access, and Health Policy

### 8.1. Acquisition and Administration Costs

Ocrelizumab has a much higher acquisition price than rituximab, especially since rituximab biosimilars were introduced. For example, Croatian maximum permitted medicine prices (Mediately; accessed October 2026) list a 600-mg maintenance infusion of ocrelizumab at approximately €11,421.66, twice the €5710.83 price of the 300-mg formulation. Rituximab is listed at approximately €2083.66 for the initial 1000-mg dose and €1041.83 for each subsequent 500-mg dose given every six months, which matches the regimen evaluated in OVERLORD-MS. The first-year acquisition cost is therefore approximately €3125.49 for rituximab and €22,843.32 for ocrelizumab. In each maintenance year, it is approximately €2083.66 versus €22,843.32. This large price gap is the main reason why rituximab is probably the more cost-effective treatment for multiple sclerosis. These figures represent drug acquisition only and do not include administration, monitoring, or other healthcare costs.

### 8.2. Cost-Effectiveness and Remaining Uncertainty

A second reason is that the two drugs probably differ little in major long-term clinical outcomes. If those differences are small, they may not offset a large gap in acquisition prices. This is consistent with the treatment-sequence model by Smets et al. In that model, the cost-effectiveness of rituximab relative to ocrelizumab depended mainly on rituximab achieving disability outcomes comparable to those of first-line high-efficacy therapies [44].

That assumption cannot yet be verified directly. OVERLORD-MS provides randomized evidence of non-inferiority for MRI disease activity in a selected early relapsing population, but it does not establish equivalence in long-term disability outcomes [23]. Cost-effectiveness must therefore still be estimated using models that link MRI activity to disability accumulation, treatment persistence, safety, quality of life, productivity, and caregiver burden [44,45,46]. The uncertainty around those assumptions should be explored explicitly in sensitivity analyses [47].

### 8.3. Health-System Context and Access

Cost-effectiveness and access also vary across healthcare systems. Drug prices, procurement arrangements, biosimilar uptake, infusion capacity, monitoring costs, and the consequences of relapse and disability differ by country, with acquisition cost often carrying greater weight in resource-constrained and low- or middle-income settings. Administration should also be included in future comparisons: intravenous rituximab and intravenous ocrelizumab require infusion capacity, premedication, and monitoring, whereas subcutaneous ocrelizumab may alter chair time and healthcare-resource utilization [8]. Country-specific health technology assessment is therefore preferable to assuming that one cost-effectiveness estimate is universally transferable [48].

## 9. Future Directions: From Drug Comparison to Treatment Strategy

Substantial comparative evidence is now available for rituximab and ocrelizumab, but future studies are still needed to determine whether differences in molecule, regimen, regulatory status, and price translate into clinically meaningful differences across patient populations and long-term outcomes.

The new direct comparative evidence narrows one important uncertainty in newly diagnosed relapsing MS. However, it does not establish that the treatments are equivalent across disease phenotypes, treatment histories, dose schedules, or long-term safety outcomes. Future trials should make these boundaries explicit. Pragmatic studies could compare fixed-interval dosing, extended intervals, and biomarker-guided re-treatment, but they need outcomes that matter to patients as well as MRI. Relapses, disability, serious infections, immunoglobulin trajectories, vaccine responses, treatment discontinuation, patient-reported burden, and health-system costs should be collected together rather than in separate research silos.

Biomarker work also needs a higher standard. Peripheral B-cell counts and subsets are promising tools for understanding immune reconstitution, but association is not enough. A useful biomarker must be analytically reliable, improve prediction beyond routine clinical and MRI assessment, and change management in a way that improves outcomes. Studies should test whether a proposed threshold works in an external cohort before using it to guide redosing. They should also avoid treating surrogate immune measures as direct evidence of disease control or clinical protection.

The anti-CD20 field is becoming more diverse. Ofatumumab and ublituximab illustrate that CD20-directed treatment can differ in route of administration, antibody engineering, and treatment experience [49,50]. Their phase 3 trials were not designed to directly compare them to other CD20 antibodies, so there is room for a broader anti-CD20 comparison study in the future.

Comparative effectiveness should therefore lead to more precise treatment decisions, rather than a broad assumption that anti-CD20 agents are interchangeable. The next studies need to show who can safely receive less treatment, who needs closer monitoring, and which trade-offs are acceptable to patients and health systems. A low-cost off-label therapy may be a rational access strategy without being identical to a licensed alternative in every respect. Conversely, a licensing history does not answer the affordability question.

That work should be planned with implementation in mind. Registries, trial extensions, and administrative data can complement one another if they use compatible definitions for exposure, infections, immunoglobulin outcomes, disability, and treatment discontinuation. Patients should help define which outcomes deserve priority. A small difference in MRI activity may matter less to some patients than the ability to complete a vaccination schedule, avoid repeated travel for infusions, or retain access to a high-efficacy therapy.

## 10. Conclusions

Current comparative evidence narrows—but does not eliminate—the distinction between rituximab and ocrelizumab in MS. OVERLORD-MS shows that, in previously untreated adults with early active relapsing MS receiving the studied regimens, rituximab is non-inferior to ocrelizumab for the prespecified MRI endpoint. It does not establish equivalence for relapses, disability progression, long-term safety, pregnancy, PPMS, previously treated patients, more diverse populations, or alternative and extended dosing strategies. Rituximab’s substantially lower acquisition cost may broaden access, whereas ocrelizumab retains the advantages of an MS-specific licence and a broader randomized development programme. The two agents should therefore be considered closely related but not universally interchangeable; selection should integrate disease phenotype, prior treatment, safety and reproductive context, dosing and monitoring strategy, regulatory status, patient preference, and healthcare-system constraints.

## Figures and Tables

**Table 1:** Pivotal trials for rituximab and ocrelizumab.

Trial	Population and n	Design and Regimen	Primary Endpoint	Principal Result/Boundary
HERMES [2]	Relapsing-remitting MS; n = 104	Phase 2, double-blind; rituximab 1000 mg IV on days 1 and 15 vs. placebo; 48 weeks	Total gadolinium-enhancing brain lesions at weeks 12, 16, 20, and 24	Rituximab reduced gadolinium-enhancing lesions and the proportion with relapses through 48 weeks. Not designed for long-term or uncommon safety outcomes.
OLYMPUS [14]	Primary progressive MS; n = 439	Randomized, double-blind, placebo-controlled; two 1000-mg rituximab infusions every 24 weeks through week 96	Time to 12-week confirmed disability progression	The primary endpoint was not significant in the overall population. Prespecified subgroup findings require cautious interpretation.
OPERA I/II [4]	Relapsing MS; n = 821 and n = 835	Two phase 3 trials; ocrelizumab 600 mg IV every 24 weeks vs. interferon beta-1a 44 micrograms SC three times weekly; 96 weeks	Annualized relapse rate	Ocrelizumab had lower annualized relapse rates and less MRI activity than interferon beta-1a. This was not a rituximab comparison.
ORATORIO [5]	Primary progressive MS; n = 732	Phase 3, 2:1 randomized; ocrelizumab 600 mg IV vs. placebo every 24 weeks for at least 120 weeks	12-week confirmed disability progression	Ocrelizumab reduced 12-week confirmed disability progression versus placebo. The population and comparator differ from OLYMPUS.

**Table 2:** Selected direct observational comparisons of rituximab and ocrelizumab.

Study/Population	Treatment Exposure or Regimen	Follow-Up	Main Finding and Interpretative Boundary
Roos et al. [15]; MSBase + Danish MS Registry; matched OCR n = 710, RTX n = 186	Routine-practice dosing was heterogeneous; sensitivity analysis restricted to OCR 600 mg every 6 months and RTX ≥ 1000 mg every 6 months.	Mean pairwise-censored follow-up 1.4 years.	RTX had higher relapse activity (ARR ratio 1.8; relapse HR 2.1); disability accumulation did not differ. Observational design and variable RTX dosing limit causal interpretation.
Chu et al. [16]; British Columbia RMS; clinical analysis n = 538	Routine-practice RTX and OCR regimens; propensity-score matched 1:1.	Common clinical comparison window: 2 years.	Very low relapse rates in both groups (ARR 0.05 RTX vs. 0.03 OCR); estimates favored OCR but confidence intervals were wide and comparisons were underpowered by rare relapses.
Savolainen et al. [17]; Finnish MS Registry; matched n = 112 (56/56)	Routine-practice RTX and OCR treatment in two university hospitals.	Median 3.1 years RTX and 2.6 years OCR.	ARR 0.03 in both groups; relapse-free survival, MRI activity, adverse events, and IgG trajectories were broadly comparable. Sample size was modest.
Frisell et al. [19]; nationwide Swedish cohort; 9704 RTX and 148 OCR treatment starts	Registry-defined treatment starts; real-world dosing and sequencing, with many OCR starters previously exposed to RTX.	Mean follow-up 5.4 years RTX and 4.7 years OCR.	Infection and relapse risks were similar between RTX and OCR, although both had more infections than other DMTs. Marked imbalance in exposure numbers and treatment sequencing complicate interpretation.
Cerono et al. [20]; University of California target-trial emulation	UCSF: OCR n = 542, RTX n = 271; UC-wide validation: OCR n = 486, RTX n = 162. RTX dose varied (median ~2 g/year at UCSF).	Multi-year retrospective follow-up in two independent health-system cohorts.	RTX was associated with higher all-cause hospitalization and hypogammaglobulinemia. Residual confounding, dose heterogeneity, and healthcare-utilization ascertainment remain important limitations.

**Table 3:** Comparative safety evidence and remaining uncertainty.

Domain	Randomized/Direct Evidence	Real-World or Supporting Evidence	Interpretation
Infections	OVERLORD-MS: ≥1 infection in 82% RTX vs. 69% OCR; serious adverse events 8% vs. 7% [23].	Swedish nationwide cohort: similar infection risk [19]; UC cohorts reported more hospitalization with RTX [20].	A possible difference in common infections remains unresolved; the randomized trial was not powered for safety.
Hypogammaglobulinemia	OVERLORD-MS: 0% RTX vs. 2% OCR; very few events [23].	UC cohorts found higher risk with RTX [20]; meta-analyses show cumulative Ig decline with anti-CD20 therapy [24,25].	Exposure duration, dose, baseline IgG and prior therapy are important confounders; monitor IgG with either agent.
Infusion reactions	Switching study: 14% in both groups over 24 weeks [26].	FAERS did not show a clear differential infusion-reaction reporting signal [27].	No convincing comparative advantage has been established.
Malignancy	One melanoma in the OCR arm of OVERLORD-MS; trial too small for inference [23].	No direct comparative study has sufficient exposure to establish a differential malignancy signal [28,29,30].	Rare-event uncertainty remains substantial.
Pregnancy/infant outcomes	OVERLORD-MS excluded pregnancy [23].	OCR: prospective MINORE/SOPRANINO and MS cohorts [31,32]. RTX: non-MS registries plus MS-specific cohort data [33,34].	Evidence is asymmetric and regulatory contraception intervals differ (OCR 4 months; RTX 12 months) [8,13].

## Data Availability

No new data were created or analyzed in this study. Data sharing is not applicable to this article.

## Abbreviations

The following abbreviations are used in this manuscript:

ADCC	Antibody-dependent cellular cytotoxicity
ADCP	Antibody-dependent cellular phagocytosis
CDC	Complement-dependent cytotoxicity
CDR-H3	Complementarity-determining region heavy chain 3
COVID-19	Coronavirus disease 2019
EDSS	Expanded Disability Status Scale
FAERS	Food and Drug Administration Adverse Event Reporting System
FcγRIIIa	Fc gamma receptor IIIa
HTA	Health technology assessment
HTAR	Health Technology Assessment Regulation
IgG	Immunoglobulin G
IRR	Infusion-related reaction
MRI	Magnetic resonance imaging
MS	Multiple sclerosis
MS4A1	Membrane spanning 4-domains A1
PPMS	Primary progressive multiple sclerosis
POMS	Pediatric-onset multiple sclerosis
RMS	Relapsing multiple sclerosis
RRMS	Relapsing-remitting multiple sclerosis
T2	Transverse relaxation time 2
Th1	T helper type 1
Th17	T helper type 17
